# The Association between Subjective and Objective Financial Knowledge: Path Analysis to Investor Behavior by Age

**DOI:** 10.1093/geroni/igab046.3290

**Published:** 2021-12-17

**Authors:** Rain Lee, Jin Sil, Euijin Jung

**Affiliations:** 1 Yeshiva University, New York, New York, United States; 2 UC San Diego, San Diago, California, United States; 3 University of Kansas, Kansas, Kansas, United States

## Abstract

Financial literacy affects stock market participation, as well as individuals’ age, gender, income, and education level. However, measuring financial literacy is more appropriate to identify individuals with strong knowledge of finance rather than average individuals with general knowledge. This could be problematic to identify general participation of the stock market and investment as more individuals are now participating without having to have such knowledge. This study explored how individuals’ subjective financial skills and well-being affect investment participation by age. Overall, males are likely to participate more in both retirement and non-retirement investment. In between the boomer generation and younger group, the younger generation who reported participating in a non-retirement investment, such as stock market were having a higher score on subjective financial well-being (STDYX = .052, 95% CI [.07, .08]; p < .05). Importantly, among the older group, subjective financial skill score becomes a predictor of participating stock market (STDYX = .09, 95% CI [.01, .17]; p < .05). As the result suggest, while younger participants focus more on financial well-being, such as having security on finances, when they are participating in a non-retirement investment, whereas older adults are likely to invest based on their beliefs on financial skills regardless of secured finances. A retirement plan has shifted toward less on savings and more on investing. Older adults are now interested more in participating in investments, such as the stock market than the young population, and the proper preparedness for those older adults in participating in the investment is needed.

